# The persistence of legal uncertainty on EU citizens’ access to social benefits in Germany

**DOI:** 10.1177/09589287221095028

**Published:** 2022-05-16

**Authors:** Angie Gago, Constantin Hruschka

**Affiliations:** 27213 University of Lausanne, Lausanne, Switzerland; 28316 Max Planck Institute, Munchen, Germany

**Keywords:** Legal uncertainty, EU law, Court of Justice of the European Union, national courts, EU citizens, social benefits

## Abstract

Legal uncertainty may hinder the effective implementation of public policies. Still, the political and legal dynamics that underpin its persistence are underexplored. This article proposes that legal uncertainty is more likely to persist in multi-level political and legal systems where actors with authority on the same issue hold different interpretations of rules. Also, it suggests that, under these conditions, actors can use legal uncertainty as an opportunity to advance their own interests. We illustrate this argument by investigating the legal uncertainty concerning EU citizens’ access to social benefits in Germany. Through the analysis of social legislation and courts’ rulings, the article shows that different interpretations of EU law by domestic actors hindered the possibilities of settling uncertainty: national courts of different levels used litigation processes and referrals to the Court of Justice of the European Union to advance their legal interpretations and the German government profited from the uncertainty to exclude EU citizens from social benefits.

## Introduction

Legal uncertainty is a universal result of law-making processes that has consequences for the formulation of public policies (Schmidt, 2008: 300; Beck, 2016: 484). Uncertainty is understood here as the lack of predictability of certain rules that might hinder the implementation of policies (Schmidt, 2008: 300). However, even though we know much about how legal uncertainty emerges and how to resolve it (Beck, 2016: 485; Betlem, 2002), the political and legal conditions that underpin its persistence have been less researched. Which political and legal dynamics can hinder its resolution? Under which conditions does legal uncertainty prevail?

In this article, we suggest that legal uncertainty is more likely to persist under the political and legal dynamics of ‘constitutional pluralism’ (Sweet and Stranz, 2012): where there is a diversity of political and legal actors with legal authority on the same issue that hold different policy choices and interpretations of rules. The article illustrates this argument by analysing the persistence of legal uncertainty regarding the access to social benefits by economically inactive EU citizens in Germany. With social benefits we refer to means-tested social assistance benefits contained in the Social Code Books II and XII. The access of EU citizens to these social benefits in Germany has been at the forefront of political and legal debates since the transposition of Directive 2004/38/EC, which aimed at regulating residence and social benefit’s entitlements of EU nationals in other EU member states.

Our country-case study is a ‘most likely’ or critical case study (Flyvbjerg, 2006: 231) to test our theoretical expectations because the formulation of EU citizens’ social benefits entitlements is conducted in a context of two-level ‘constitutional pluralism’ (Sweet and Stranz, 2012) where multiple authorities can decide at different levels. The first is the supranational level of the EU that regulates EU free movement and the access of economically inactive EU citizens through Directive 2004/38/EC, social security regulations and Court of Justice of the European Union (CJEU) case-law. The second one is the national level where governments are still responsible for social policies and courts ensure compliance with law. Besides, in the German legal system, there exist social courts and regional courts that might lead to the emergence of conflicting lines of case-law.

We already know that legal uncertainty on EU citizen’s social benefits entitlements originated at the supranational level. In the run-up to the Eastern Enlargement of 2004, member states could not achieve a political compromise to regulate the access of economically inactive citizens to social benefits. This led to the inclusion of ambiguous provisions in Directive 2004/38/EC (Blauberger and Schmidt, 2014: 2–3). Also, this uncertainty was reinforced by the existence of further contradictions between several legislative provisions and previous judgments of the CJEU such as the *Grzelczyk* case (C-184/99) (Gago and Maiani, 2022: 262; Blauberger and Schmidt, 2014: 2). As a result, the transposition of Directive 2004/38/EC into national law became difficult for domestic actors. Governments, national courts and administrations had to navigate through a complex system of supranational and national rules that was opened to interpretation (Blauberger and Schmidt, 2014: 2–3).

This article takes this argument forward and investigates the political and legal dynamics that have underpinned the persistence of legal uncertainty since the approval of Directive 2004/38/EC, which has not yet been researched. To do so, the article uses qualitative case study techniques and process-tracing to reconstruct the German historical social policy and legal processes from the enactment of the Directive 2004/38/EC to the last ruling of the CJEU on those issues in 2020. In a second step, we test our arguments by collecting ‘multiple independent observations’ such as documentary sources such as parliamentary debates, government reports, CJEU case-law, national legislation and media news (Beach and Pedersen, 2013: 128). This technique known as ‘triangulation’ allows us to avoid the problem of ‘unreliable measures’ (Beach and Pedersen, 2013: 128).

Empirically, the article contributes to the literature by showing that the interaction between EU law and national political and legal dynamics opened three streams of legal uncertainty in Germany. The first stream refers to the use of alternative sources of EU law, other than the Directive 2004/38/EC, to grant social benefits to economically inactive EU migrants. The second stream refers to the testing of the limits of the Directive 2004/38/EC, and the third stream refers to the exploration of the relationship between EU law and national constitutional law.

Theoretically, the article shares new theoretical knowledge on the different implications of legal uncertainty for domestic actors. Previous studies have shown that legal uncertainty is considered by administrations as an obstacle (Blauberger and Schmidt, 2017: 914). Instead, this article adds new knowledge on *how* governments and national courts can use legal uncertainty as an opportunity to advance their own policy options and interpretations (Schmidt, 2008: 300).

We show that national courts of different levels used litigation processes and referrals to the CJEU to advance their legal interpretations. The Federal Social Court (FSC) used legal uncertainty to advance an expansive position that aligned the EU norms with German constitutional case-law to guarantee the access of EU nationals to minimum subsistence. In parallel, social courts and regional social courts took different positions and supported different sides in the legal conflict between the legislator and the FSC.

At the political level, the German legislator, in agreement with other domestic actors such as lower courts and municipalities, attempted to exploit to a maximum the opportunities afforded by Directive 2004/38/EC to restrict in practice access to such benefits and to reduce resulting costs and public discussions. As a result, government interventions have not necessarily contributed to resolving all the streams of legal uncertainty in Germany.

This article is organized as follows: the second section explains how legal uncertainty regarding EU citizen’s access to social benefits emerged in the context of the creation of Directive 2004/38/EC, reviews the literature on how domestic actors can respond to legal uncertainty and introduces our theoretical expectations. The third section offers the empirical analysis. The last section concludes.

## Legal uncertainty on EU citizen’s social benefits entitlements: Origins and persistence

The theoretical focus of this article is to analyse the political and legal dynamics that might reinforce legal uncertainty but, first, it is important to reflect upon what we understand by legal uncertainty and how the latter can originate. By legal uncertainty, we refer to the lack of predictability of certain rules (Schmidt, 2008: 300). In this sense, we follow Schmidt’s understanding of legal uncertainty because, from a political science perspective, we are interested in the implications that the lack of predictability can have on the formulation and implementation of public social policies (Schmidt, 2008: 300; Beck, 2016: 484).

Moreover, legal uncertainty is understood here as a ‘natural’ and universal consequence of law-making that usually originates in ‘primary legal materials’ such as treaties (Beck, 2016: 484). This might be due to the ambiguity of the text (Beck, 2016: 484) or lack of information concerning general principles (Dequech, 2001: 914) between others. Regarding our case study, previous studies have already shown the importance of these factors (ambiguity, lack of information and so on) to explain the origins of legal uncertainty on EU citizen’s social benefits, as we are going to see in the next section.

### The origins of legal uncertainty and Directive 2004/38/EC

We can distinguish two strands of literature that have studied the origins of legal uncertainty on EU citizen’s social benefits. The first strand of the literature has focused on analysing the ambiguity and lack of information of certain provisions of Directive 2004/38/EC to explain the origins of legal uncertainty. Directive 2004/38/EC was negotiated between member states and the European Commission in the context of the 2004 Eastern Enlargement and after the two landmark rulings of the expansive phase of the CJEU (Gago and Maiani, 2022: 265).

In 1998, the Court decided in the *Martinez Sala* judgment (C-85/96) that EU citizens who apply for social assistance benefits could appeal to the non-discrimination principle of the Article 12 of EC Treaty. And, in 2001, in the *Grzelczyk* case (C-184/99) the CJEU appealed to equal treatment to grant benefits to ‘lawfully residing Union citizens’ (Blauberger et al., 2018: 1425), which meant that member states could not limit the access to social benefits to EU nationals. In this context, member states negotiated Directive 2004/38/EC and some member states wanted a restrictive text that gave more leeway to limit social rights of EU citizens due to ‘fears of welfare tourism’ (Gago, 2021: 209). However, due to the general compromise character of EU law-making processes, Directive 2004/38/EC ended up containing ambiguous provisions that led to ‘significant legal uncertainty’ (Blauberger and Schmidt, 2014: 2).

In matters of welfare access, Directive 2004/38/EC established a clear distinction between two categories of EU citizens^1^: workers and persons economically inactive in the host country. Workers can have access to social benefits derived from their own social security contributions and other means-tested benefits. Instead, economically inactive EU nationals are expected to have ‘sufficient resources’ in order not to become a ‘burden’ to the financial systems of the host countries.

Also, Directive 2004/38/EC provides that when a worker becomes involuntarily unemployed, for the purpose of access to social benefits, he or she can retain the status of a ‘worker’ for up to 6 months if the person was employed for less than 1 year. If the person becomes involuntarily unemployed after more than 1 year, he or she does not lose the status of ‘worker’ or self-employed person if he or she is registered as a jobseeker with the relevant employment office (Article 7 of the Directive).

Besides, if the person is not able to prove a genuine link with the local labour market and to demonstrate active search for employment in the host state, s/he may eventually lose the possibility of receiving social assistance benefits. Only after a period of 5 years of residence, do EU migrants have access to a permanent residence permit that fully secures equal access to social assistance benefits regardless of nationality or labour market status (Article 16 of the Directive). In addition, Article 24 (2) of the Directive gives some discretion to the member states when it provides that states are ‘not obliged’ (under EU law) to pay social assistance to economically inactive persons, giving states room to pay or not.

Legal ambiguity emerged because several provisions of Directive 2004/38/EC indeed left ‘considerable room for interpretation’ because it employs vague concepts, such as ‘sufficient resources’, ‘genuine activities’, ‘real link’ or ‘unreasonable burden’ (Blauberger and Schmidt, 2014: 2–3). Directive 2004/38/EC did not contain specific information about what sufficient resources or unreasonable burden means, for example. Finally, there was tension between certain provisions of Directive 2004/38/EC with case-law of the CJEU existing at that time (Blauberger and Schmidt, 2014; Gago and Maiani, 2022).

On the other hand, a second strand of the literature argues that legal uncertainty is the result of the over-constitutionalization of the EU Treaties. With over-constitutionalization Grimm refers to the process whereby the CJEU has ‘constitutionalized’ EU Treaties. According to Grimm, this process removes important principles that should be contained in national ordinary law from political decision-making and the representative sphere (Grimm, 2015: 460). In so doing, the CJEU might preclude governments to rectify uncertainty through national legislations.

This view has been contested by other authors such as Davies who argues that over-constitutionalization does not necessarily lead to de-politicization because national courts and legislature can challenge the interpretations of the CJEU (Davies, 2018: 359). The discussions on over-constitutionalization are relevant for our case study because some studies have demonstrated that there are indeed conflicts between general principles contained in the EC Treaties that can generate legal uncertainty. For example, Martinsen has analysed the lack of clear boundaries between the non-discrimination principle and the principle of proportionality (Martinsen, 2011: 944).

### The persistence of legal uncertainty: Political and legal dynamics at the national level

As we have seen above, the origins of legal uncertainty have been extensively studied by the literature. But we still do not know much about the conditions under which legal uncertainty persists. In this article, we suggest that legal uncertainty is more likely to persist in a context of ‘constitutional pluralism’ (Sweet and Stranz, 2012: 97) where a multiplicity of policy preferences and legal interpretations of the same rules are likely to emerge. In the case of EU citizen’s social benefits, we can talk about a two-level constitutional pluralism. The first (supranational) level refers to the EU where constitutional pluralism produces a legal and political system with authorities operating at different positions (Sweet and Stranz, 2012: 97).

Within the EU’s legal order, social policies remain in the domain of member states, while at the same time, the latter are bound to respect and implement the rules regulating the free movement of EU citizens and the principle of non-discrimination. The potential conflict between the norms at the EU level and the existence of national social policies that generally fall within the competency and responsibility of member states (Blauberger and Schmidt, 2014: 2; Roos, 2016: 270) has led the CJEU to give authoritative judgments on the definition of EU migrants’ social rights. The second (national) level refers to the functioning of the German legal system that is characterized by the existence of the legislator and of social courts and regional courts that might lead to conflicting lines of case-law.

Moreover, this article proposes that domestic actors can use legal uncertainty as an ‘opportunity structure’ that serves them to ‘pursue their private interests’ (Schmidt, 2008: 300). Although national actors might not seek legal uncertainty strategically, the latter allows for ‘more room for politics in EU implementation processes’ (Martinsen et al., 2019: 827), and there is more space for governments to test the limits of EU law (Gago and Maiani, 2022).

First, governments are responsible for transposing EU directives into national law, but there is a certain margin of manoeuvre about how to transpose. The implementation of EU law is ‘in fact the locus where an appropriate practical balance can be found between supranational authority and national autonomy, legal compliance and contextual discretion’ (Ferrera, 2014: 826). In the case of convergence of policy choices, governments can exploit the provisions of EU regulations to advance their policy prerogatives. But, even in cases of divergence, national governments can also use the leeway of legal uncertainty to advance their choices. One way to do that is to ‘contain compliance’ with EU law (Conant, 2002) to keep their regulatory preferences to a certain extent. Another way to use the window of opportunity of legal uncertainty is to ‘push the boundaries of EU law and to promote, in dialogue with the Court, a change of jurisprudence that reflects their agendas and preferences’ (Gago and Maiani, 2022: 271).

The second relationship that we need to consider is the one between national actors, in particular governments, and the CJEU. Member states are obliged to comply with the decisions of the CJEU by re-adjusting national practice as well as the interpretation of the respective norms to the rulings of the Court. But some studies have shown that legal uncertainty can amplify the policy space of governments to maintain their national policy preferences vis-à-vis the Court’s rulings (Wasserfallen, 2010: 1134). Therefore, we expect governments to profit from legal uncertainty because they can use it to support their policy choices.

Furthermore, to explain the persistence of legal uncertainty it is important to look beyond the relationship between European regulations, CJEU case-law and member states’ governments and parliaments (Blauberger and Schmidt, 2017: 911) and look at the role of national courts. When there is legal uncertainty, we assume the emergence of different tensions depending on the type of interaction between national courts with 1) the CJEU, 2) national governments and 3) other national courts operating at different levels.

One strategy available for national courts vis-à-vis legal uncertainty originated by EU law is to refer a question of interpretation of EU law to the CJEU to clarify a legal question that is decisive in a case before the national court. After this preliminary reference procedure, national courts have to decide on the individual case. Legally, national courts must – as all national actors – comply with the CJEU’s authoritative interpretation of EU law. However, as the CJEU only gives a ruling on a question of legal interpretation, the practical implementation depends on the national court. This sometimes leads to the impression that these questions are settled depending on national judges’ choices (Sweet and Stranz, 2012: 96).

From a legal point of view, when there is divergence between national court practice and a CJEU ruling, national courts have to apply national law in line with the ruling of the CJEU. In practice, national courts will therefore not openly rule against a CJEU ruling, but may use remaining legal uncertainty as a strategy to circumvent this obligation. Despite its limits, this procedure gives power and even political autonomy to national courts. In cases where their legal interpretation is aligned with the CJEU’s, they might be interested to use the preliminary reference to validate their interpretations vis-à-vis national governments or other actors.

National courts may in certain cases use this procedure to challenge national policies (Tridimas and Tridimas, 2004), to influence the executive and the legislative or even oblige or incite them to change national legislation (Golub, 1996: 362). The decision of the courts can be seen as messages that are used in ‘policy struggles and thus contribute to shaping the outcomes of political decision making’ (Bonjour, 2016: 329).

The same strategy may also be used when there are disagreements between national courts at different levels. They can use referrals to the CJEU to validate their legal interpretation and to settle disagreements between higher and lower national courts (Alter, 1996: 470). As a result, the multiplication of courts competent in this area leads inter alia to the situation that some German courts self-consciously use the supranational level, the CJEU, to enhance their own authority (Sweet and Stranz, 2012: 97). Therefore, we expect that legal uncertainty gives opportunities for national courts to validate their reasonings and interpretations. The authoritative nature of rulings of the CJEU may in the case of divergence be used as a tool to advance their legal interpretations.

## The transposition of Directive 2004/38/EC in Germany

Directive 2004/38/EC was transposed in 2004 in the *Freedom of Movement Act/EU 2004* that included the regulations regarding the right of entry and residence of EU migrants. Section 4 of the Act is dedicated to non-economically active migrants. It notes that they need to have sufficient own resources, including health insurance to be entitled to settle in the host country. Moreover, in 2006, the first government of Angela Merkel, a coalition between the Christian Democratic Union (CDU) and the Social Democratic Party (SPD), introduced changes to the Social Code Book II (SCB II). The system of social assistance benefits in Germany was transformed after the so-called *Hartz IV* reforms, implemented in 2005, which divided social assistance benefits into two groups: social benefits for generally employable persons, the Social Code Book II (SCB II), and basic minimum subsistence benefits for persons who are unable to support themselves by means of gainful employment, other sources of income (an example being passive income through real estate, among others), the Social Code Book XII (SCB XII), which are paid by municipalities. Both are means-tested benefits that can be accessed if a person legally resides in Germany (Bruzelius, 2019: 77), although there are exceptions for Germans who reside abroad (SCB XII, Section 24).

The subsequent changes provided by the *Social Security Act 2006* led to the exclusion of newly arriving EU nationals from the social assistance benefits of the SCB II. Since then, national legislation refuses social assistance benefits to both EU citizens benefiting from the freedom of movement for the first 3 months of their stay in Germany in general, and for persons ‘who are in Germany solely for the purpose of finding gainful employment from the basic insurance for unemployed’.^2^ As we expected theoretically, the government exploited to a maximum the opportunities afforded by Directive 2004/38/EC to restrict access to social assistance benefits. These new provisions of the 2006 Act seemed to comply with the Directive 2004/38/EC, but there were doubts on the compatibility of Article 24 of Directive 2004/38/EC with the non-discrimination Articles 12 and 39 of the EC Treaty and national regulations. In 2007, the Social Court in Nuremberg referred questions for a preliminary ruling in the case of *Vatsouras* (C-22/08 and C-23/08) to clarify this.

In its ruling in 2009, the Court considered that an unemployed person may not be excluded from social benefits if those benefits are aimed at facilitating the access to the labour market. The respective possibility to exclude certain EU nationals from social assistance provided for by Article 24 (2) of the Directive does not apply to such benefits. In so doing, the Court did not provide a ‘clear standpoint’ (Hailbronner and Thym, 2013: 28).

### The unfolding of legal uncertainty: The first two streams

The latter ruling showed that Directive 2004/38/EC ‘leaves considerable scope for the judiciary to interpret the actual meaning of the legislative text’ (Martinsen, 2011: 951). This led to different levels of German national courts interpreting EU law and national regulations differently, leading to a ‘fair amount of divergent national jurisprudence’ (Devetzi, 2019: 341). The first *stream of uncertainty* relates to uses of alternative sources of EU and international law rather than Directive 2004/38/EC. National courts of different levels supported their divergent decisions by resorting to different EU legal acts as well as other norms of international law.

For example, on 19 October 2010, the FSC ruled (No. B 14 AS 23/10R) that the exclusion of a French jobseeker from social benefits violated the European Convention of Social and Medical Assistance (ECSMA). The ECSMA was established by the Council of Europe in 1953 and provides that ‘parties undertake to ensure that the nationals of other parties, who are lawfully present in their territory and who are without sufficient resources, are entitled to the same social and medical assistance as their own nationals’ (Council of Europe, 1953: 1). To solve the conflict between national social policy regulations and the ECSMA, the German government added a reservation to the Convention in 2011 that suspended equal treatment of foreign nationals in this field. Consequently, access to the Basic Social Assistance (SCB XII), to the Basic Income Support for Jobseekers (SCB II) as well as to benefits falling under the Federal Social Assistance Act were no longer obligatory under ECSMA.

In parallel, however, a *second stream of uncertainty* emerged in which *the limits of the Directive 2004/38/EC were tested.* The divergent jurisprudence raised doubts regarding the scope of the right to free movement and its limits regarding access to social benefits. In a judgment of 30 January 2013 (No. B 4 AS 54/12R), the FSC reversed a judgment by the Regional Social Court of Baden-Württemberg that had denied the entitlement to social benefits resulting from SCB II to a Bulgarian citizen as the claimant did not fall within the scope of the Directive 2004/38/EC. The FSC used the notion of ‘habitual resident’ as an alternative legal reasoning to justify its decision and argued that the pregnant Bulgarian claimant, who was habitually resident in Germany, had a right to stay because of her being in the process of founding a family (with a Greek national residing in Germany).

According to the FSC, it could not be proven that her residency ‘resulted solely from the purpose of seeking employment’ (Federal Social Court, 2013). However, lower courts resisted the 2013 FSC ruling (Blauberger and Schmidt, 2014: 5) and in some cases deviated from it (Absenger and Florian, 2018: 164). Building on earlier judgments of the Regional Social Courts of Lower Saxony-Bremen and of Berlin Brandenburg, the Berlin Social Court, for example, criticized the FSC for disregarding the intention of the legislator by resorting to the SCB XII that is only applicable to persons who are unfit to work on a permanent basis.^3^

The ruling of the FSC – and its consequence that an alternative right to stay may circumvent the exclusion of unemployed EU migrants from the access to social benefits under SCB II – was heatedly contested by a significant number of German local authorities that are responsible for providing social welfare benefits to EU nationals (Heindlmaier and Blauberger, 2017: 1198). Some studies showed that certain cities were under financial pressure due to the uneven geographical distribution of EU migrants. In Germany, most of EU migrants reside in Berlin and the large cities of the former West Germany (Bruzelius et al., 2015: 410).

Some municipalities, which are responsible for assessing whether EU migrants are eligible to claim social benefits (Heindlmaier and Blauberger, 2017: 1211) and to pay the benefits contained on the SCB XII, declared that from 2007 to 2012 the number of Romanians and Bulgarians had increased from 30,000 to 70,000 (Roos, 2016). This led to a rise of the expenditures for social benefits for certain municipalities. In turn, several cities (Berlin, Duisburg, Dortmund and Mannheim) asked the Federal Government for financial help (DW, 2013a). Against this background, local authorities and social courts intensified the effort to define under which circumstances a right to stay may entitle persons to benefits under the SCB II.

### The first political response

The political campaign of the municipalities intensified pressure on the government to act. In a position paper, they openly asked the government to take more restrictive measures (German Association of Cities, 2013). However, at that moment, the federal government held a more ‘nuanced position’ (Hailbronner and Thym, 2013: 15) and did not see that EU migration created a problem for the cities (Deutscher Bundestag, 2013). The government opted to relieve the financial strain of the local authorities but did not enact new legislation to settle uncertainty. It increased the resources of the European social fund and the Fund for European aid to the most deprived by €200 million (Bruzelius et al., 2015: 412). Also, the federal government transferred further funds (€25 million) to municipalities and increased its contribution to the costs for accommodation and heating, which are provided to EU migrants under SCB II. Additionally, the government appointed an inter-ministerial Committee on Labour and Social Affairs to decide on further measures. The final report of the State Secretaries Committee^4^ contained recommendations on changes to the legislation aimed at tightening EU migrants’ entitlement to social benefits.

These measures were not implemented in the short-term, however. The government was reluctant to further tighten the legislation due to the empirical evidence that there was not a generalized problem of welfare migration in Germany. From 2007 to 2015, the percentage of economically inactive EU migrants who accessed basic unemployment benefit under SCB II had increased only by 1.89 per cent, and EU citizens receiving *Sozialhilfe*^5^ amounted only to 0.81 per cent (Martinsen and Werner, 2019: 648*).* Another reason that explains the policy inertia of the government was that the CDU and SPD shared a lack of electoral interest to politicize EU migration because their constituencies are traditionally divided on immigration issues (Franzmann et al., 2020: 632).

Nevertheless, the politicization of the access of EU migrants to social benefits increased during the electoral campaign for the European Parliamentary elections in 2014. A new right-wing political party formed in 2013, the so-called Alternative for Germany (AfD), placed claims of welfare migration of Eastern European migrants at the centre of its political agenda with the slogan ‘kick out welfare cheaters’ (Arzheimer, 2015: 554). In addition, the CSU asked the government for actions to avoid ‘poverty migrants’ coming from Bulgaria and Romania (DW, 2013b). Also, part of the German media contributed to this narrative by claiming that migration from Eastern Europe posed ‘a major risk to the economy’, mostly in relation to the ethnic ‘Roma’ community (Bruzelius et al., 2014: 17).

As a result of these pressures, the government introduced new changes in the *Freedom of Movement Act/EU 2014* to allow the use of temporary re-entry bans in cases of fraud related to the right of free movement. The new legislation also limited the residence right of EU jobseekers and lowered it to the minimum required by EU law (6 months). This helped to clarify some legal questions regarding the access to social benefits of EU jobseekers but did not settle the second stream of uncertainty.

### The persistence of legal uncertainty: Protracted legal disagreement

As we expected theoretically, national courts used referrals to the CJEU to advance their legal interpretations. Legal disagreement between the courts continued, and both the FSC and lower courts decided to turn the definitional matter to the CJEU for resolution (Blauberger et al., 2018: 1437; Devetzi, 2019: 342). In 2013, the Social Court of Leipzig referred questions for a preliminary ruling to the CJEU; in the case of *Dano* (Case C-333/13), a Romanian national who had been economically inactive throughout her residence in Germany, and who had been denied social assistance benefits by the Jobcentre Leipzig.

In 2014, the FSC referred questions for another preliminary ruling to the CJEU; in the case of *Alimanovic* (Case C-67/14), a Swedish national who after being unemployed for 1 year had lost her status as a worker and was denied social assistance benefits by the Jobcentre Berlin-Neukölln. In both cases, the German government took the position that the respective claimants were not entitled to social benefits as they were not entitled to benefits under the Directive 2004/38/EC.

Finally, the CJEU settled the second stream of legal uncertainty by confirming the position of the German government and by referring lower national courts in the *Dano* and *Alimanovic* cases. The CJEU fully supported the exclusion of economically inactive citizens from social assistance benefits in both cases in 2014 and 2015, respectively. This restrictive line was confirmed in the *Garcia Nieto* case (C-299/14). Therefore, the legislator saw no necessity to amend either Section SCB II or Section 23 SCB XII as it seemed that the matter was settled, by excluding non-workers as well as economically inactive jobseekers from social assistance under German law.

### The third stream: German constitutional law

Divergent jurisprudence, however, continued and created a *third stream of legal uncertainty* consisting of *the exploration of the relationship between EU law and national constitutional law*. On 3 December 2015, the FSC followed the CJEU’s decision in the *Alimanovic* case and argued that there was no entitlement to social benefits under the SCB II for persons not covered by the Directive 2004/38/EC. Even so, the FSC resorted to an alternative legal reasoning: the FSC found that the discretion contained in the legal provision of the SCB XII contradicted Article 1 of Germany’s Basic Law (human dignity principle) as well as its Article 20 (welfare state principle), and decided to grant at least minimum benefits to persons in need.^6^ This case proves what we expected according to our theory, that national courts can use the preliminary procedures to the Court to challenge national policies.

This third stream adheres to a traditional legal value of the German Basic Law that provides that all persons staying on German soil have a right to social aid if they are in need. Nonetheless, lower courts did not share this analysis and adhered to the reasoning of the legislator that the provision of social assistance may be subject to conditions. The legal reasoning behind the position of lower courts is that in the context of free movement in the EU, it is not unreasonable to require persons to return to his or her country if they cannot provide for themselves in the host country.

### Second government intervention

This last ruling of the FSC on this matter in 2015 led again to increasing concerns by local authorities about their capacity to limit EU migrants’ social benefits (DW, 2016b). In turn, in spring 2016, the government presented a draft legislation that aimed at resolving legal uncertainty on the matter (German Bundestag, 2016). The Act to Regulate the Claims of Foreign Persons 2016 bans the access of EU nationals to subsistence benefits if they do not meet the definition of a person entitled to free movement. In line with the Directive 2004/38/EC, this ban is supposed to exclude benefits to such persons for a period of 5 years following their entry (Fernandes, 2016: 19). Once more, the government used legal uncertainty to advance their policy options and restrict to the maximum the access of EU citizens to social benefits. The law was implemented in December 2016 after a very short legislative process that started in September 2016. It provides that: ‘The EU national, who is not working in Germany, is not self-employed or has no claim under the Social Code Book II on the basis of previous work, is not entitled to any permanent benefits under Social Code Book II and XII, within the first 5 years [of their stay]’.

Although the government noted that this reform was to resolve uncertainty (German Bundestag, 2016), other political pressures influenced the restrictive outcome of the reform. The so-called refugee crisis,^7^ had raised a political discourse based on anti-immigration sentiments and Euroscepticism (Stockemer et al., 2020). Although there was not an increase of extreme anti-immigration and Eurosceptic positions among the German citizens (Stockemer et al., 2020: 901), the question of how to deal with the refugee crisis brought about fierce political debates from within the government (largely led by the CSU) and from parts of the political opposition (mainly by the AfD). This debate was largely focused on matters of asylum and immigration by third-country nationals (TCN) and influenced perceived welfare migration debates.

In this conflict, the role of the CSU was opposed to the official position of its sister party, the CDU, to manage the refuge crisis, and strengthened the political strategy against EU welfare migration. On 2 March 2015, the former CSU MP, Stephan Mayer, participated in an event organized by the euro-critical think tank ‘Open Europe’ in the UK where he said that the facts showed ‘an increasing problem of welfare abuse by EU migrants’ (Briggs, 2015). Contrary to the CDU and the SPD, the CSU had electoral motivations to politicize the immigration issue (Franzmann et al., 2020: 618).

Moreover, the success of AfD in certain federal states traditionally governed by the social democrats ‘led to changing party-voter alignments and a strong politicization of immigration issues’ (Franzmann et al., 2020: 614). In Hamburg, for example, the SPD had won the 2015 state elections with 48.4 per cent of the votes but without absolute majority. Both parties running the federal government, the SPD and the CDU, lost seats, four and eight, respectively, whereas for the first time since its establishment, the AfD was elected to the Hamburg state parliament with 6.1 per cent of the votes, corresponding to eight seats (DW, 2015).

In January 2016, inspired by the measures taken by the UK during the run-up to Brexit, the former SPD vice president and former major of Hamburg, Olaf Scholz (who is currently Germany’s Chancellor), demanded that the government should put in place further social policy restrictions for EU migrants (DW, 2016a). The rest of SPD’s federal leadership sided with Scholz’s demands. This was also the case of the Minister of Labour, Andrea Nahles (SPD), who became the most visible supporter (also due to her position in the government) of the proposal to ban the access of EU migrants to social benefits for 5 years. Nahles justified the measures ‘to avoid significant financial strains on cities that come with such social benefits’ (DPA/The Local, 2016).

The 2016 legislation largely settled the *second stream of legal uncertainty* as the definitions and timelines were clear and access to social assistance for EU migrants was limited to the minimum required by the Directive 2004/38/EC. In this regard, the legislator and its allies in the immigration authorities and lower courts had been able to advance their policy preferences using the perceived uncertainties of European law. However, as the next section shows, the two other streams are still unresolved, and decisions by immigration authorities as well as local and regional courts are still very diverse and partially contradictory.

### A new turn to the first stream

In 2020, the German government amended again the *Freedom of Movement Act/EU* with the main objective being to adapt legislation according to the new situation of the UK after Brexit. The *Act on the Current Amendment of the Freedom of Movement Act/EU* was adopted on 9 October 2020 and entered into force on 24 November 2020 with the main objective being to protect the status rights of UK nationals and their family members entitled to freedom of movement in line with the Withdrawal Agreement.^8^

The reform also brought about new discussions on welfare migration. The initial proposal of this Act contained an amendment that would have led to an obligation of immigration authorities to examine the right of residence of EU citizens in line with the Directive 2004/38/EC, with binding effect on the competent welfare authorities. This decision was taken because the government considered that German welfare authorities are ‘too generous’ (Rath, 2020a). However, on 6 October 2020, only 1 day after the expert hearing, and days before the parliamentary debate, the latest judgment of the CJEU (Case C-181/19) provided that national authorities could no longer reject social benefits to EU migrants automatically if their right to reside didn’t derive from Directive 2004/38/EC. This led to the decision to drop the amendment. The expert hearing, as well as consultations with relevant NGOs and associations considered that immigration authorities would not be technically prepared or in a position to individually examine the welfare situation of EU citizens in line with the legal requirements because their administrative practice is often too restrictive (Rath, 2020b).

The above ruling of the CJEU referred to the case *Jobcenter Krefeld v JD*. In February 2019, in agreement with the lower court (Social Court Düsseldorf), the Higher Social Court of North Rhine-Westphalia referred the case *Jobcenter Krefeld v JD* to the CJEU for a preliminary ruling claiming that the right of residence under Article 10 of Regulation No. 492/2011 (interpreted as granting a residence right to parents taking care of children enrolled in a school in another member state) would not fall within the scope of the Directive 2004/38/EC and would therefore not be suspended if the person loses the status of a worker.

The case C-181/19 concerned a Polish national who had lived in Germany since 2012 with his two daughters and had been refused social assistance benefits under SCB II, subsidiary unemployment benefits and social allowances for the two daughters. The Jobcentre Krefeld ‘rejected that application based on point 2 of the second sentence of Paragraph 7(1) of the SCB II, on the ground that JD [the claimant] had not retained the status of a worker and that he was residing in Germany solely to seek employment’.

The Social Court of Düsseldorf annulled the rejection and ordered the Jobcentre Krefeld to pay the benefits applied for. It argued that the residence right derived not from Directive 2004/38 but from Article 10 of Regulation No 492/2011 for minor children and parents caring for them. Nevertheless, the Jobcentre brought an appeal against that judgment and the case was subsequently referred to the CJEU, which confirmed the ruling of the Social Court of Düsseldorf and the opinion of the Regional Social Court. The ruling of the CJEU questioned relevant German legislation and argued that the exclusion laid down in point 2(c) of the second sentence of Paragraph 7(1) of the SGB II, in that it entails the ruling that nationals of other member states who have a right of residence based on Article 10 of Regulation No 492/2011 are categorically and automatically denied any entitlement to the subsistence benefits at issue in the main proceedings, is contrary to Article 4 of Regulation No 883/2004.

Therefore, the CJEU decided that national authorities cannot *automatically* exclude EU migrants from social assistance benefits even if their right of residence does not derive from the Directive 2004/38/EC. The CJEU reasoned that member states have an obligation to check *individually* if the right to reside of a Union citizen derives from other regulations (e.g. Regulation No. 492/2011), prior to denying access to social benefits. With this case, the first stream of legal uncertainty (using alternative legal sources to the Directive 2004/38/EC) seems to be settled, favouring the legal interpretation of the FSC and other Social Courts at the expense of the government and the legislator.

As the issue was settled by the CJEU, the government had no option to disregard its reasoning. This situation may lead to further legal uncertainty as in the second stream, the restrictive government position prevailed, while in the first stream, it was the FSC’s legal position that was confirmed by the CJEU. Considering current developments, it is highly likely that legal uncertainty will still prevail once the third stream is settled. Another case relating to the question of the entitlement to social benefits under Germany’s Basic Law for otherwise excluded EU citizens remains pending with the German Federal Constitutional Court (GFCC) and local and regional practice is diverse on this issue.

## Conclusions

This article has shown how legal uncertainty on EU nationals’ access to social benefits in Germany, which emerged due to the ambiguity of Directive 2004/38/EC and the openness of Article 24(2), was later reinforced by national political and legal dynamics. After the German legislator had decided in 2006 to restrict access to social assistance to EU migrants, a legal and political debate began that led to legal uncertainty regarding the limits of this exclusion.

The article identifies three streams of legal uncertainty in this regard. The first stream refers to the use of alternative sources of EU law, other than the Directive 2004/38/EC, to grant social benefits to economically inactive EU migrants. The legal interpretations as well as the related policy choices of the actors on this were divided between the more expansive legal position of the FSC that had on several occasions identified alternative legal sources for an entitlement to social benefits, and the more restrictive positions of the government and most of the lower courts. The last ruling of the CJEU (2020) settled this stream in favour of the more expansive legal interpretation of the FSC by arguing that member states cannot automatically exclude EU migrants from social benefits if their right to reside does not derive from the Directive 2004/38/EC.

The second stream refers to the testing of the limits of the Directive 2004/38/EC. This stream was settled by the government through the 2016 reform that responded to the restrictive policy objectives of the legislator and corresponding interpretations by lower courts and that denies access to such benefits for 5 years. The third stream refers to the exploration of the relationship between EU law and national constitutional law. The FSC has attempted to overcome the restrictive outcome regarding the second stream by opening a new debate on the compatibility of the exclusion of vulnerable EU migrants with German constitutional law. This stream is still open and new developments thereof may also lead to a reopening of the first two streams.

It is important to consider the profits of legal uncertainty in the case of Germany: if legal uncertainty prevails, governments and authorities at the local and regional level retain a greater level of discretion in terms of the scale and scope of application of policies. The article shows that legal uncertainty widens policy options for the government that can choose how to respond through polity inertia or restrictive legislation, depending on competition between political parties and electoral dynamics. Additionally, we have demonstrated that the possibility to have some margin of manoeuvre in the application of legislation regarding social benefits in general also reflects the divergence of the federal states in this regard. Taking away this leeway, by clarifying the legal standards as binding upon all authorities may be seen as detrimental to the federal system and its diverse actors.

## Notes

1 The EU also approved Regulation 883/2004 on the coordination of social security systems that focuses on contributory benefits for workers.

2 Act amending the Second book of the Social Code (2007) – Section 7 (1)(2) SCB II.

3 Sozialgericht Berlin, judgment of 11 December 2015, S 149 AS 7191/13.

4 ‘Legal issues and challenges in claiming of the social security systems by members of the EU member states.’

5 Benefits available for persons incapable of working, with reduced earnings capacity or of old age.

6 Bundessozialgericht, judgment of 3 December 2015, B 4 AS 44/15R.

7 Like in the case of welfare migration, the use of the term ‘refugee crisis’ reflects the use of the notion by politicians and the ruling parties.

8 Agreement on the withdrawal of the United Kingdom of Great Britain and Northern Ireland from the European Union and the European Atomic Energy Community (OJ 2019/C 384 I/01).
